# Metabolic signature associated with parameters of the complete blood count in apparently healthy individuals

**DOI:** 10.1111/jcmm.14383

**Published:** 2019-06-19

**Authors:** Annette Masuch, Kathrin Budde, Gabi Kastenmüller, Anna Artati, Jerzy Adamski, Henry Völzke, Matthias Nauck, Maik Pietzner

**Affiliations:** ^1^ Institute of Clinical Chemistry and Laboratory Medicine University Medicine Greifswald Greifswald Germany; ^2^ German Centre for Cardiovascular Disease (DZHK e.V.), partner site Greifswald Greifswald Germany; ^3^ Institute of Bioinformatics and Systems Biology Helmholtz Zentrum München Neuherberg Germany; ^4^ Institute of Experimental Genetics, Genome Analysis Centre Helmholtz Zentrum München Neuherberg Germany; ^5^ Lehrstuhl für Experimentelle Genetik Technische Universität München Freising‐Weihenstephan Germany; ^6^ DZD (German Centre for Diabetes Research) München‐Neuherberg Germany; ^7^ Institute for Community Medicine University Medicine Greifswald Greifswald Germany; ^8^ DZD (German Centre for Diabetes Research), site Greifswald Greifswald Germany

**Keywords:** apparently healthy, blood cell metabolism, complete blood count, metabolomics, population‐based study

## Abstract

Metabolomics studies now approach large sample sizes and the health characterization of the study population often include complete blood count (CBC) results. Upon careful interpretation the CBC aids diagnosis and provides insight into the health status of the patient within a clinical setting. Uncovering metabolic signatures associated with parameters of the CBC in apparently healthy individuals may facilitate interpretation of metabolomics studies in general and related to diseases. For this purpose 879 subjects from the population‐based Study of Health in Pomerania (SHIP)‐TREND were included. Using metabolomics data resulting from mass‐spectrometry based measurements in plasma samples associations of specific CBC parameters with metabolites were determined by linear regression models. In total, 118 metabolites significantly associated with at least one of the CBC parameters. Strongest associations were observed with metabolites of heme degradation and energy production/consumption. Inverse association seen with mean corpuscular volume and mean corpuscular haemoglobin comprised metabolites potentially related to kidney function. The presently identified metabolic signatures are likely derived from the general function and formation/elimination of blood cells. The wealth of associated metabolites strongly argues to consider CBC in the interpretation of metabolomics studies, in particular if mutual effects on those parameters by the disease of interest are known.

## 1 INTRODUCTION

The complete blood count (CBC) is often used in the general evaluation of a person's health and is one of the most commonly ordered clinical laboratory tests in medical patients. It has the potential, when interpreted carefully and in association to the clinical state, to provide useful information to assist in diagnosis and management. The CBC includes both quantitative evaluation of erythrocytes, leukocytes and platelets as well as detection of morphological abnormalities that provide useful insights to various disease conditions.

In healthy adults, leucocyte (white blood cells, WBC), erythrocyte (red blood cells, RBC) and platelet (PLT) count depends on many different factors. WBC count is influenced by age, gender, health status (eg trauma, infections, sepsis, age‐related diseases etc), environmental factors, genetic inheritance, stress level, diet and lifestyle (eg chronic psychological stress).^1^, ^2^, ^3^, ^4^ To characterize erythrocytes several parameters are commonly measured in CBC evaluations: RBC count, haemoglobin (HGB), haematocrit (HCT), mean corpuscular volume (MCV), mean corpuscular haemoglobin (MCH), mean corpuscular haemoglobin concentration (MCHC) and red cell distribution width (RDW). Typically, RBC, HGB and HCT are closely related and analysed as an entity, while RDW is generally analysed in conjunction together with MCV and RBC.^5^ The main function of platelets (PLT) is to contribute to primary haemostasis and thrombosis formation. Furthermore, they have different roles in inflammation, atherosclerosis, angiogenesis, antimicrobial host defense, and contribution to wound healing.^6^, ^7^, ^8^, ^9^ Mean platelet volume (MPV) is described as a marker of platelet reactivity^10^ and platelet function.^11^

Previously, we have examined the metabolic signature of low‐grade inflammation including the WBC count and identified interesting associations with plasma lactate and pyruvate levels translating experimental findings to the general population.^12^ To further extend the knowledge about metabolites associated with blood cells and their indices, all main parameters of the CBC should be subjected to metabolomics investigation.

As part of the typical routine analysis and usually done in first line examinations CBC is widely used to diagnose medical conditions like anaemia, blood loss, thrombocytopenia, acute and chronic infections, abnormalities in blood cells, allergies, leucocythemia and other blood related diseases.^13^, ^14^, ^15^ Changes in CBC accompany also diseases like leucemia,^16^ hepatitis^17^ or hypertension.^18^ Besides, CBC is typically included in population‐based studies while metabolomics research is increasingly expanding to large study samples. In line with this, RDW has been identified in recent years as prognostic marker for cardiac, non‐cardiac as well as all‐cause mortality in a variety of diseases.^19^ Likewise, MPV was suggested as prognostic biomarker for cardiovascular risk.^10^ Thus, if specific diseases are examined on the basis of a metabolomics study, changes in metabolites that associate to changes in CBC parameters independent of disease may lead to misinterpretation. Therefore, we intend to examine the metabolic signature associated with parameters of the CBC in a comprehensive manner among an apparently healthy population excluding the influence of severe disease.

## 2 MATERIALS AND METHODS

### 2.1 Study population

The Study of Health in Pomerania (SHIP‐TREND) is a population‐based study representative for West Pomerania, a rural region in north‐east Germany.^20^ A stratified random sample of 8826 adults aged 20‐79 years was drawn from population registries. Sample selection was facilitated by centralization of local population registries in the Federal State of Mecklenburg‐West Pomerania. Stratification variables were age, sex and city/county of residence. Baseline examinations were conducted between 2008 and 2012. Out of all invitations 4420 choose to participate (50.1% response).

The first 1000 participants from SHIP‐TREND without self‐reported diabetes mellitus type 2 who underwent a whole‐body magnetic resonance imaging; a more extensive phenotyping was performed including for example, additional laboratory measurements and metabolome analyses. This most comprehensively analysed sub‐sample of SHIP was chosen to ensure a maximum of available clinically relevant information.

Out of these 1000 subjects those with missing values in one or more parameters of the CBC or confounders considered (N = 7), those reporting intake of antithrombotic agents (N = 65; ATC code B01A) or anaemia treatment (N = 10, ATC code B03A and B03B) or who presented with an acute inflammatory state or missing high‐sensitivity C‐reactive protein (hsCRP) levels (N = 39; hsCRP >10 mg/dL) were excluded resulting in a study population of 879 subjects.

### 2.2 Ethical approval and informed consent

All participants gave written informed consent before taking part in the study. The study was approved by the ethics committee of the University of Greifswald and was performed in accordance with the principles of the declaration of Helsinki.

### 2.3 Laboratory measurements and phenotypic characterization

Smoking status (current, former or never smokers), daily alcohol consumption, physical activity (≥1 hour training a week) and educational attainment (<10, 10 or >10 years of school) were assessed using computer‐aided personal interviews. Anthropometric measurements were taken during the physical examination. Height was measured to the nearest 1cm using a digital ultrasound instrument. The body weight of the participants was conducted in light clothes and without shoes using standard digital weight scales. Body‐mass‐index (BMI) was calculated as weight (kg)/height^2^ (m^2^). Mean daily alcohol consumption was calculated using beverage‐specific pure ethanol volume proportions. Fasting blood samples were taken from the cubital vein of participants in the supine position between 7.00 am and 12.00 am All samples were either analysed immediately or stored at −80°C. Serum cystatin C, lipids (total cholesterol, high‐density (HDL) and low‐density lipoprotein (LDL) cholesterol, triglycerides) and serum activities of alanine amino transferase (ALT) were measured by standard methods (Dimension VISTA, Siemens Healthcare Diagnostics, Eschborn, Germany). Cystatin C‐based eGFR was calculated using the CKD‐EPI equation.^21^ CBC parameter and indices were analysed in EDTA whole blood samples using the Sysmex XT 2000 Haematology Autoanalyser (Sysmex, Kobe, Japan) according to the manufacturer’s recommendation. Throughout the study period, two levels of quality control material (QC‐XN‐Check Level 1 and QC‐XN‐Check Level 2; Streck Laboratories Inc, Omaha, NE, USA) were performed for all routine parameters to control the performance of the overall procedure.

### 2.4 Metabolomics measurements

#### 2.4.1 Metabolomics measurements based on MS

A detailed description of measurement techniques has been published elsewhere.^12^, ^22^ Briefly, non‐targeted metabolomics analysis for metabolic profiling was conducted at the Genome Analysis Centre, Helmholtz Zentrum München, Germany. Two separate LC‐MS/MS analytical methods were used as previously published^23^ to obtain broad metabolite spectra in plasma samples in a non‐targeted manner. After pre‐processing, 475 plasma metabolites remained for the statistical analyses. Note that 177 metabolites could not be unambiguously assigned to a chemical identity and are referred to hereafter with the notation ‘X’ followed by a unique number. Targeted metabolomics profiling of the same plasma samples was performed using the AbsoluteIDQ p180 Kit (BIOCRATES LifeSciences AG, Innsbruck, Austria) and was conducted at the Institute of Clinical Chemistry and Laboratory Medicine, University Medicine Greifswald, Germany. This approach allows simultaneous absolute quantification of 188 metabolites using a combination of liquid chromatography (Agilent 1260 Infinity Binary LC, Santa Clara, CA, USA) and mass spectrometry (AB SCIEX 5500 QTrap™ mass spectrometer; AB SCIEX, Darmstadt, Germany). After normalization and pre‐processing of the data, 183 metabolites were available for subsequent analysis.

### 2.5 Data integration

Most of the metabolites were unique to one of the applied techniques. However, 44 plasma metabolites were overlapping with both techniques. Following the grouping of metabolites in biochemical classes (ie lipids, amino acids and carbohydrates), correlations of those metabolites measured on both platforms were computed with all members of the same biochemical class. Subsequently, the metabolite with the higher median correlation across all class members was kept for further analysis. In total, 613 plasma metabolites were used in the subsequent statistical analyses.

### 2.6 Statistical analysis

Linear regression models were performed to assess the associations between CBC parameters (ie HGB, HCT, MCV, MCH, MCHC, RBC, RDW, PLT, MPV and WBC; continuously) with plasma metabolite levels (dependent variables; continuously). To this end metabolite levels were log‐transformed. Sex and smoking behaviour were used as categorical confounders. Age, BMI, serum ALT and eGFR were used as continuous confounders. Notably, men and women were combined in the present analyses as no strong evidence for an interaction between sex and one of blood cell measures became obvious in regression analyses. Correction for multiple testing was done using the Benjamini‐Hochberg procedure, controlling the false discovery rate (FDR) at 5%. Dependencies between metabolites were reconstructed using a Gaussian graphical model (GGM) following previous work.^24^ Statistical analyses were performed using R 3.3.2 (R Foundation for statistical computing, version 3.3.2, Vienna, Austria).

## 3 RESULTS

### 3.1 Population characteristics

Men showed a less favourable health status compared to women, including for example, an unfavourable lipid profile and higher alcohol consumption (Table 1). With the exception of MPV, all blood cell measures showed the expected sexual dimorphism (Table 1). Most obvious differences were seen in HB (higher in men) and PLT (higher in women). However, this dimorphism did not translate into the association patterns with respect to metabolites. Hence, we combined both sexes in the following analyses.

**Table 1:** General characteristics of the study population by sex

Characteristics	Men (N = 388)	Women (N = 491)	*P* a
Age, y	49 (38; 59)	49 (40; 59)	0.43
Smoking, %			
Never	32.9	50.1	<0.01
Former	44.0	27.7	
Current	23.1	21.2	
Education, %			0.05
<10 y	9.5	11.8	
10 y	53.6	59.1	
>10 y	36.9	29.1	
Alcohol consumption, g/d	8.55 (2.98; 17.96)	2.25 (0.71; 5.52)	<0.01
BMI, kg/m^2^	27.3 (25; 29.8)	25.9 (23; 29.3)	<0.01
Ferritin, µg/L	1.48 (0.84; 2.45)	0.58 (0.27; 1.01)	<0.01
Transferrin, g/L	2.5 (2.2; 2.7)	2.6 (2.3; 2.9)	<0.01
White blood cell count, Gpt/L	5.27 (4.58; 6.21)	5.49 (4.78; 6.45)	0.04
eGFR, mL/min/1.73 m^2^	118 (109; 125)	113 (105; 121)	<0.01
HDL‐cholesterol, mmol/L	1.28 (1.11; 1.47)	1.58 (1.35; 1.83)	<0.01
LDL‐cholesterol, mmol/L	3.4 (2.77; 4.01)	3.35 (2.76; 4)	0.71
Erythrocyte count, Gpt/L	4.9 (4.6; 5.1)	4.4 (4.2; 4.7)	<0.01
Haemoglobin, mmol/L	9.15 (8.8; 9.5)	8.3 (7.9; 8.6)	<0.01
Haematocrit	0.44 (0.42; 0.45)	0.40 (0.38; 0.42)	<0.01
MCV, fL	90 (87; 92)	90 (88; 93)	0.03
MCH, fmol	1.88 (1.83; 1.94)	1.86 (1.81; 1.92)	<0.01
MCHC, mmol/L	21 (20.6; 21.3)	20.6 (20.3; 20.9)	<0.01
RDW, %	13 (12.6; 13.4)	13.1 (12.6; 13.6)	0.03
PLT, Gpt/L	208 (183; 236)	234 (204; 272)	<0.01
MPV, fL	10.2 (9.7; 10.8)	10.3 (9.7; 10.9)	0.59

Note

Data are expressed as median (25th; 75th percentile). To convert haematocrit to %‐values multiply by 100.

Abbreviations: ALT, alanine aminotransferase; BMI, body‐mass‐index; eGFR, estimated glomerular filtration rate; HDL, High‐density lipoprotein; LDL, Low‐density lipoprotein.

a Wilcoxon‐rank‐sum test for continuous and chi‐squared test for categorical data was used for comparison.

### 3.2 Associations between blood cell measures and plasma metabolites

**Figure 1:**
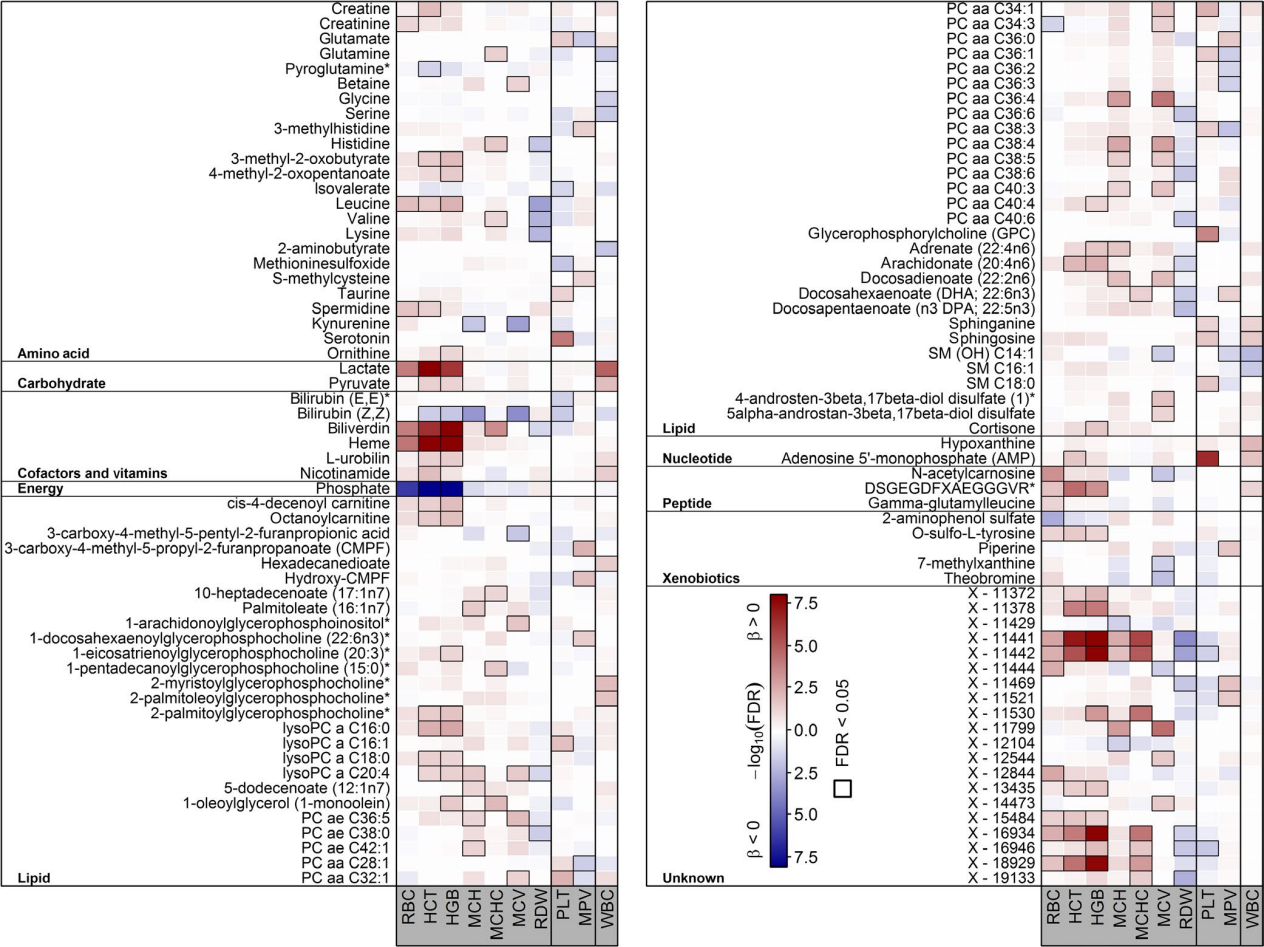
Heat map of corrected *P*‐values (controlling the false discovery rate [FDR] at 5%) from linear regression analyses using one of the red blood cell count traits as exposure and metabolites as outcome adjusting for age, sex, waist circumference, smoking, serum alanine aminotransferase activities and estimated glomerular filtration rate. Orange shading indicates positive and blue shading indicates negative associations, respectively. HCT, haematocrit; HGB, hemoglobin; MCH, mean corpuscular hemoglobin; MCHC, mean corpuscular haemoglobin concentration; MCV, mean corpuscular volume; MPV, mean platelet volume; PLT, platelets; RBC, red blood cells; RDW, red cell distribution width; WBC, white blood cells

**Figure 2:**
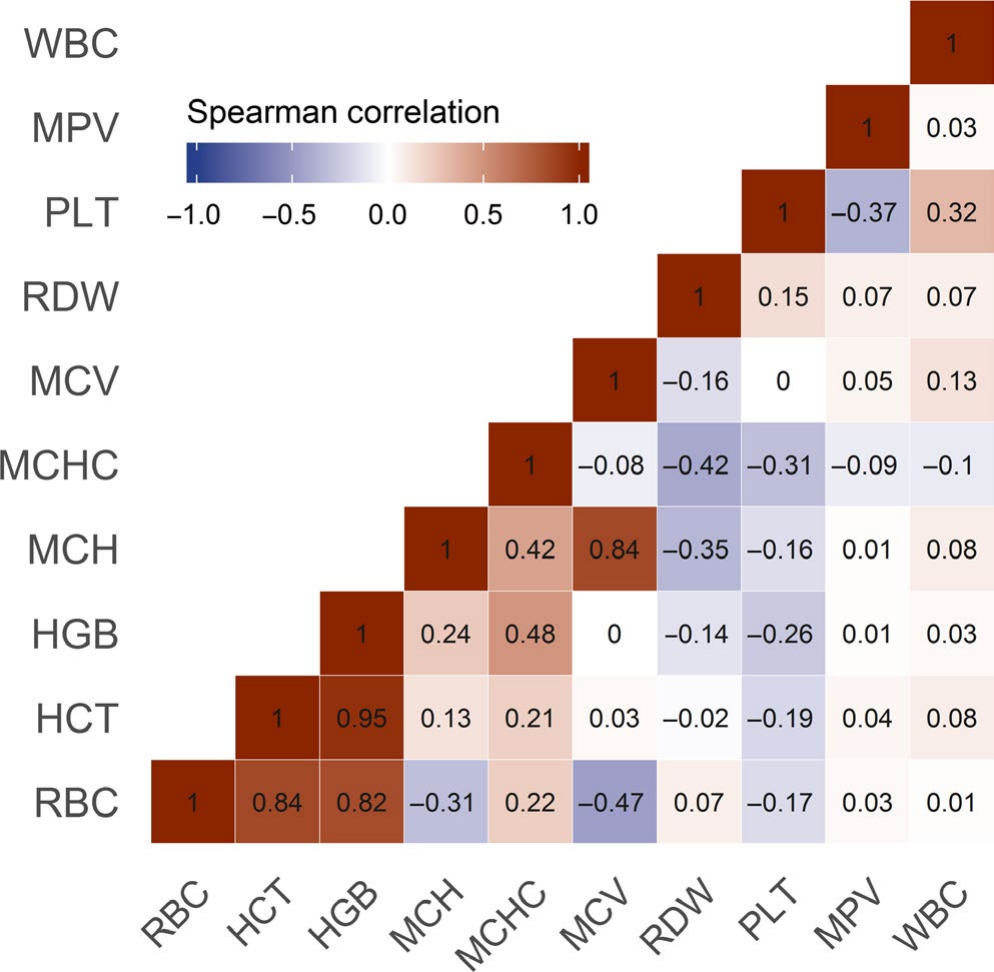
Correlation matrix of the complete blood count derived measures. Numbers within the cells indicate Spearman correlation coefficients. RBC, red blood cells; HCT, haematocrit; HGB, haemoglobin; MCH, mean corpuscular haemoglobin; MCHC, mean corpuscular haemoglobin concentration; MCV, mean corpuscular volume; MPV, mean platelet volume; PLT, platelets; RDW, red cell distribution width; WBC, white blood cells

**Figure 3:**
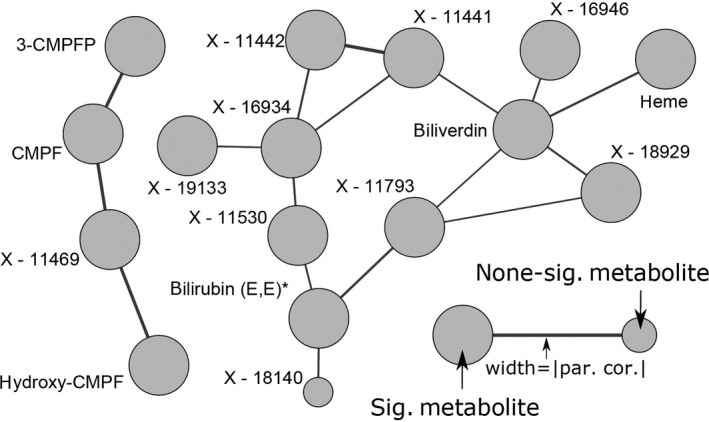
Subnetwork from the Gaussian graphical model to reconstruct metabolite dependencies with a particular focus on heme and 3‐carboxy‐4‐methyl‐5‐propyl‐2‐furanpropanoate (CMPF) related metabolites. Increased node size indicates significant associations with at least one blood cell count trait under investigation. par. cor., partial correlation

In total, 118 metabolites significantly associated with at least one of the CBC measures under investigation (Figure 1). HGB and HCT were the strongest traits with 35 and 31 associated metabolites, respectively. As expected from their close correlation (Figure 2), almost all associations consisted of the same metabolites. For example, strong positive associations with metabolites of heme degradation, branch‐chained amino acids (BCAA), lysophosphatidylcholines (lysoPC), lactate or arachidonate became obvious (Figure 1). Notably, several of the positively associated unknown compounds clustered around heme in the derived GGM (Figure 3). Inverse associations with HGB/HCT were limited to phosphate, bilirubin (Z,Z) and pyroglutamine. Despite RBC shared many of the associations seen with HCT and HGB, unique positive associations with N‐acetylcarnosine, gamma‐glutamylleucine and creatinine became obvious.

Even MCH and MCV showed highly similar association patterns comprising positive associations with lysoPC C20:4, several phosphatidylcholines (PC; eg PC ae C36:5, PC aa C36:4 or PC aa C40:3), docosadienoate and some unknown compounds. Inverse association with both parameters comprised kynurenine, bilirubin (Z,Z), theobromine and one unknown compound.

With the exception of biliverdin and likely related unknown compounds (Figure 3), MCHC showed a distinct metabolic profile compared to the other red blood cell measures, comprising positive associations with glutamine, histidine, valine, 10‐heptadecanoate, 1‐monoolein, docosahexanoate and one unknown compound (Figure 1). RDW showed inverse associations with a number of the already named amino acids (eg, leucine, histidine or valine), ω3‐polyunsaturated fatty acids (ω3‐PUFA), three diacyl PCs as well as several unknown compounds.

Significant opposing associations with PLT and MPV were seen for glutamate, PC aa 36:1 and PC aa 38:3. Strong positive associations with serotonin, adenosine‐5`‐monophosphate (AMP) and glycerophosphorylcholine were unique to PLT, similar as taurine, lysoPC C16:1, two PCs, sphingosine and the sphingomyelin SM C18:0. Inverse association with PLT could be noted for isovalerate, methioninesulfoxide and some unknown compounds. MPV was positively associated with 3‐methylhistidine, 3‐methylcysteine, 3‐carboxy‐4‐methyl‐5‐propyl‐2‐furanpropanoate (CMPF) and related unknown compounds (Figure 3). Furthermore, several PC species, for example, PC aa C36:2 and PC36:3, and sphingomyelins, for example, SM(OH) C14:1, were inversely associated with MPV only.

Despite some different exclusion criteria, WBC associations replicated those from our previous publication^12^ and were in part mirrored by PLT or MPV.

## 4 DISCUSSION

This study examined the metabolic signature associated with parameters of the CBC. Overall, 118 metabolites were associated with at least one of the CBC parameters. Strongest associations were observed with metabolites of heme degradation as well as phosphate and lactate. Although RBC is typically analysed in combination with HCT and HGB to gain plausible results for diagnosis, such functional grouping was mirrored only to a certain extent within associated metabolites. Similarly, RDW, reflecting the degree of variation in size of RBC, is determined and analysed together with MCV and RBC but the observed metabolic signature did not reveal interrelated associations. In contrast, MCV and MCH showed closely related associations. For PLT the associations were either opposing to those observed for MPV or they were unique to either PLT or MPV. Notably, this study clearly distinguishes from our previous work on inflammatory parameters, including WBC.^12^

### 4.1 RBC energy production

Plasma lactate levels were one of the strongest positive associations seen with RBC and related measures HCT and HGB. RBC lack mitochondria and hence rely on glucose supply from the circulation for energy generation, that is, adenosine triphosphate (ATP) formation, by means of anaerobic glycolysis like the Embden‐Meyerhof pathway.^25^ The coenzyme nicotinamid adenine‐dinucleotide (NAD^+^) serves as acceptor of the electron released during ATP production in glycolysis. Through conversion of pyruvate NADH is reduced and NAD^+^is regenerated under production of lactate. Notably, it is well‐accepted that changes in RBC metabolism can be observed as changes in blood lactate levels.^26^ A higher amount of RBC would subsequently increase lactate production and hence explains our observation. Fittingly, pyruvate and nicotinamid showed significant positive associations with HGB and HCT, and with HCT, respectively.

Notably, the processes of binding, transport and delivery of oxygen do not require the expenditure of energy by the RBC, however, maintenance of the normal cellular function does. Energy is needed to keep the iron within the haemoglobin in its divalent form, to maintain the ion concentrations within the cell against concentration gradients, and to prevent reduction in sulfhydryl groups by enzymes.^27^ This is mirrored within the metabolic signature because one of the most prominent associations is the inverse relationship between phosphate and the parameters RBC, HGB and HCT. Phosphate is a major component in energy consumption and production, used to build up ATP in catabolic reactions for energy storage or released during hydrolyzation reactions to deliver energy. In line with this adenosine monophosphate was positively associated with HCT.

### 4.2 Heme metabolism

A major molecular signature associated with RBC and related measures comprised merely positive association with metabolites putatively related to heme degradation, for example, heme itself or biliverdin. Haemoglobin is composed of four globular protein subunits forming a structure strongly binding the heme group in which Fe^2+ ^is covalently bound within a porphyrin ring.^28^ Degradation of the heme group, for instance from aged erythrocytes, gives rise to bile pigments. Biliverdin, the green bile pigment, is produced from heme upon separation of Fe^2+^ and CO. In turn, biliverdin is reduced to bilirubin, the yellowish bile pigment. Free unconjugated bilirubin is lipid‐soluble and potentially toxic, thus for excretion it has to be transported to the liver where it is conjugated to glucuronic acid, therefor it binds reversibly to albumin.^29^ Conjugated bilirubin is excreted with the bile and is converted to urobilinogen in the large intestine by bacterial proteases, part of it is excreted with the feces. Part of the urobilinogen is reabsorbed to the blood stream and is oxidized to urobilin and excreted by the kidney.^30^ Notably, based on direct neighbourhood in the GGM several yet unknown compounds showed similar positive associations likely attributing those to intermediates of heme degradation. However, bilirubin was inversely associated with RBC, HGB and HCT which was to some extend surprising. Considering the binding of bilirubin to albumin, it is likely that the observed inverse association is due to methodological artefacts. During sample preparation large proteins like albumin—including all metabolites bound to albumin—are precipitated by methanol. In turn, bilirubin is potentially cleared from the sample before spectrometric analysis. Furthermore, partial or mild lysis of erythrocytes during sample preparation might also have given rise to the positive association with heme and related metabolites.

### 4.3 RBC and amino acids

Branched‐chain amino acids (BCAA) and their metabolites namely leucine, valine, 3‐methyl‐2‐oxobutyrate, and 4‐methyl‐2‐oxopentanoate present relatively consistent positive association with red cell measures HGB and HCT, while an inverse association with RDW was observed. Typically, BCAA are interrelated with mitochondrial β‐oxidation and energy production. However, RBC are independent of mitochondrial energy supply. Rather, BCAA and other amino acids are actively transported by RBC through blood stream and, moreover, RBC actively participate in blood tissue amino acid exchange^31^ explaining the observed positive associations for BCAA and their catabolites as well as for some other amino acids, for example, lysine and ornithine. Considering the role of BCAA as metabolic marker in health and disease, it has to be noted that increased levels of circulating BCAA are typically associated with insulin resistance (IR),^32^, ^33^ while the positive association between haematologic parameters RBC, HGB and HCT with IR is also well documented.^34^, ^35^ IR is a hallmark of non‐alcoholic fatty liver disease (NAFLD)^36^ and a positive association of iron metabolism parameters with increased risk of lean NAFLD (at BMI <25) compared to obese subjects without NAFLD has been reported.^37^ Therefore it may be conceivable that positive associations of BCAA and their metabolites especially with HGB and HCT in this study might also indicate hepatic fat accumulation. Of note, the inverse association between BCAA and RDW is quite prominent. Increased RDW has been related to inflammation,^38^ and is considered a reliable prognostic marker for cardiac, non‐cardiac as well as all‐cause mortality in a variety of diseases.^19^ In terms of the pro‐inflammatory profile associated with increased BCAA, the inverse association with RDW is rather contradictory and remains to be clarified. ω3‐PUFA also showed strong inverse associations with RDW and there is evidence that the consumption of ω3‐PUFA rich diet is beneficial for several health outcomes likely by reducing inflammation.^39^ Thus, the present findings argue in favour of this hypothesis.

### 4.4 Putative surrogates of platelet activation

PLT showed distinct associations with plasma metabolites serotonin, adenosine 5′‐monophosphate (AMP), taurine and glycerophosphorylcholine being the most prominent (all positively) and in sum might be related to platelet activation or rather platelet function. Serotonin is a well‐described vasoconstrictor circulating in the blood stream which is actively taken‐up by platelets and sequestered in δ‐granules in high concentrations.^40^ Notably, when PLT are examined emphasis is typically on platelet activation. Yet, under normal physiologic condition without injury or need for clot formation a general antithrombotic state exists in the blood to keep it liquid.^26^ Thus, clot formation is normally suppressed. Adenosine diphospate is a very potent platelet agonist involved in platelet activation and secreted from δ‐granules. Under resting conditions it is rapidly hydrolyzed to AMP by platelet surface ADPases.^26^ Therefore, the strong positive association between PLT and AMP might be interpreted as intrinsic clot preventing process. Not at least, taurine was reported to influence PLT activity and aggregation.^41^ Interestingly, low PLT taurine was reported in diabetes and supplementation of taurine restored PLT dysfunction.^42^ We like to note, that the PLT associations with taurine or serotonin did not translate to urine levels of these metabolites (both *P* > 0.20) further arguing for a local effect.

### 4.5 Kidney function

Although controlling for the eGFR in statistical analyses some metabolites in plasma previously reported as surrogates for GFR associated inversely with either RBC, MCV and to a lesser extend with MCH including creatinine, N‐acetlycarnosine^43^ and kynurenine.^44^, ^45^ The hormone erythropoietin (Epo) is produced and secreted from the kidney stimulating RBC production in the bone marrow.^46^ Chronic kidney disease (CKD) is accompanied by anaemia due to reduced renal Epo production, reduced RBC lifespan as well as impaired intestinal iron absorption among others.^47^ However, CKD‐associated anaemia is characterized by normocytic and normochromic RBC while iron deficiency anaemia is typically indicated by reduced MCV/microcytosis.^47^ Nevertheless, in the present analysis the associations do not give information with respect to clinical micro/normo/macrocytosis but present the direction of associations. Thus, at the subclinical level, it might be conceivable that inverse associations with MCV may be the results of affected kidney function due to compromised Epo production in the kidneys. A similar explanation might hold for the inverse association with metabolites of caffeine (theobromine and 7‐methylxanthine). Within the renal tubules caffeine and its metabolites are heavily reabsorbed and their renal clearance depends strongly on renal output.^48^

An independent link to kidney function is given by the positive association between MPV and the uremic toxin CMPF and related unknown compounds.^49^ Production of PLT from megakaryocytes is dependent on thrombopoietin which is produced in the kidneys besides other tissues. In line with this, CMPF‐related unknown compounds showed inverse association also with PLT. Of note, CMPF is able to inhibit cellular uptake and hence activation of the thyroid hormone thyroxine^50^ which appears to influence also PLT formation and function because hypothyroidism is accompanied by increased PLT reactivity and increased MPV.^51^

### 4.6 Phospholipids

Several phospholipid species, including diacyl PCs, ether PCs and lysophosphatidylcholines (lysoPCs), were positively associated with RBC characteristics but not RBC itself. The latter point likely indicates that our observations are not solely the result of RBC‐lysis giving rise of specific phospholipid species in plasma.^52^ Rather those phospholipids relate to shifts in lipoproteins ranging from very low‐density to high‐density ones.^53^ Indeed, RBC interact with lipoproteins and may gain or lose cholesterol from low‐density lipoprotein (LDL) and high‐density lipoprotein (HDL) particles, respectively. This process is essential for determining membrane stability, the so‐called critical fluidity and therefore is a key component in maintenance of structure and function in RBC.^54^ In healthy volunteers, RBC membrane stability was directly associated with LDL‐cholesterol^55^ and an excessive increase in cholesterol content in RBC membrane proportionally increases its rigidity which in turn reduces membrane stability and deformability. This increases vulnerability of RBC for lysis in response to mechanical forces when passing through capillaries.^54^ Notably, besides being the carrier of oxygen throughout the blood stream, RBC have been identified to play significant roles in cardiovascular function and pathology. RBC actively participate in local vasodilation and regulation of vascular tone by release of NO and ATP.^56^, ^57^ Moreover, RBC appear to contribute to development of atherosclerotic plaques, particularly due to the cholesterol content in the RBC membrane and formation of reactive oxygen species from cell free haemoglobin originating from ruptured RBC.^54^ It is widely accepted that high LDL‐cholesterol concentration in plasma leads to an excess uptake of cholesterol into the RBC membrane increasing the risk for lysis and release of haemoglobin promoting peroxidation, inflammation and atherosclerotic plaque development.^58^ Within the CBC increased rigidity/reduced membrane stability of RBC is typically reflected by increased RDW which in turn is closely associated with the risk of carotid atherosclerotic plaque development.^59^ In this study most of the associations with phospholipid species were observed for RBC parameter MCV and MCH, however, not for RDW. Nevertheless, RDW is calculated from MCV and, importantly, this study was performed in apparently healthy individuals. This fact explains that the naturally occurring interaction between RBC and lipoproteins in a physiological manner can be observed within the metabolome for parameters MCV and MCH. As pathologic cases were absent, RDW is not associated with phospholipid species in this study.

### 4.7 Strengths and limitations

A specific strength of this study is the combination of a large sample size and the comprehensive profiling of the plasma metabolome using targeted and non‐targeted mass‐spectrometric approaches. To the best of our knowledge so far no other study examined the metabolomic associations with parameters of the CBC. However, there are few drawbacks due to methodical or preanalytical issues that need to be mentioned. As already pointed out, large proteins are precipitated during sample preparation. Thus metabolites like bilirubin bound to albumin may get cleared from the sample and will be missed or potentially present misleading associations. Moreover, although EDTA is the recommended anti‐coagulant for CBC and WBC differential analysis and is commonly used in everyday practice, it is known that PLT quickly change their shape from discs to spheres when collected in EDTA‐tubes consequently affecting results for MPV (up to 20%).^60^ In turn, results presented for this parameter may have limited validity.

## 5 CONCLUSION

This study reveals specific metabolic signatures associated with parameters of the CBC that are mainly associated with the function, formation or elimination of blood cells. In addition, they might implicate subclinical alterations, for example, of kidney function since blood cell formation is also interrelated with kidney function. Knowing about such signatures improves the interpretation of metabolomic study results. This data demonstrate CBC associated metabolites in a relatively healthy population without self‐reported diabetes mellitus; therefore it may form a basis for comparisons with diseased individuals or at least provide information about observable associations that might occur in large population‐based samples only due to blood cell metabolism.

## CONFLICT OF INTEREST

The authors declare that they have no conflict of interest.

## AUTHOR CONTRIBUTION

AM analysed and interpreted results and wrote the manuscript; KB performed mass‐spectrometric measurements, analysed data and helped to draft the manuscript; GK, AA, JA performed metabolomics measurements, analysed data and reviewed statistical analysis; HV substantially contributed to the concept and design of SHIP and was main leader in SHIP data acquisition, MN supervised the project and provided materials and staff to measure laboratory parameters, MP conceptualized the project, analysed population‐based data integrating metabolomics and wrote the manuscript.

## DATA AVAILABILITY STATEMENT

SHIP data are publicly available for scientific and quality control purposes. The informed consent obtained from the participants of the SHIP study does not cover data storage in public databases due to confidentially reasons. Data usage can be applied for via www.community-medicine.de, an interface provided by the host institute of the SHIP study to ensure compliance with all legislation.
